# Investigation of Nanoscale Tungsten Carbide Enhanced Surface Carbon as a Platinum Support for the Hydrogen Evolution Reaction

**DOI:** 10.3390/nano13081369

**Published:** 2023-04-14

**Authors:** Zhiwei Liu, Yang Li, Juan Fang, Qi Wan

**Affiliations:** 1 School of Energy and Environmental Engineering, University of Science and Technology Beijing, Beijing 100083, China; 2 State Key Laboratory of Environment-Friendly Energy Materials, School of Materials and Chemistry, Tianfu Institute of Research and Innovation, Southwest University of Science and Technology, Mianyang 621010, China

**Keywords:** solution combustion synthesis, nanoscale, Pt/WC, catalyst, hydrogen evolution reaction

## Abstract

Finding new supports and reducing the amount of platinum are key steps in the development of fuel cells. Herein, nanoscale WC is used as the support for a Pt catalyst, which was prepared by an improved strategy based on solution combustion and chemical reduction. After high-temperature carbonization, the synthesized Pt/WC catalyst displayed a well-distributed size distribution and relatively fine particles, which consisted of WC and modified Pt nanoparticles. Meanwhile, the excess carbon of the precursor transformed into amorphous carbon in the high-temperature process. The formation carbon layer on the surface of the WC nanoparticles had a significant effect on the microstructure of the Pt/WC catalyst, improving the conductivity and stability of Pt. Linear sweep voltammetry and Tafel plots were used to evaluate the catalytic activity and mechanism for the hydrogen evolution reaction. As compared with the WC and commercial Pt/C catalysts, the Pt/WC catalyst showed the highest activity with η10 of 32.3 mV and a Tafel slope of 30 mV·dec^−1^ towards HER in acidic solution. These studies confirm that the formation of surface carbon can increase material stability and conductivity, improving the synergistic relationships between Pt and WC catalysts, leading to an increase of catalytic activity.

## 1. Introduction

With the increasingly serious energy crisis, it is very important to develop new energy system and end dependence on oil and natural gas. Compared with traditional fossil fuels, hydrogen energy is a clean energy with high efficiency and acceptable cost. Normally, the utilization of hydrogen energy needs fuel cell technology. However, one of the key problems is finding highly active and large-surface-area electrocatalysts for fuel cells [1]. When the cathode potential excursions as high as 1.5 V, the oxidation of carbon in the catalyst layer of fuel cells occurs, which is a major reason for performance deterioration in automotive applications [2]. In addition, natural gas is used as a hydrogen source for the anode fuel, which contains a little carbon monoxide. A Pt catalyst is the most common application for the anode material, and Pt is also widely used as a catalyst for methanol oxidation reduction, but it is easily poisoned by adsorbed CO and has a high price [3]. With the development of fuel cells catalysts, the Pt-Ru catalyst has achieved significant popularity over Pt, while its relatively expensive cost has hindered the commercialization of direct methanol fuel cells (DMFCs) [4,5,6]. Through comprehensive consideration, noble metal catalysts for fuel cells would be replaced by other potential candidates with low costs and high energy conversion systems [7,8,9].

Due to the excellent catalyst activity and durability, the catalyst of platinum supported on carbon is the most widely applied for proton exchange membrane fuel cells (PEMFCs). Nevertheless, the oxidation/corrosion of carbon support occurs during the operation of PEMFCs, which degrades the performance of the catalyst and leads to the low durability of full cells [10]. Therefore, looking for new noble metal support materials and reducing the amount of noble metal have become research hotspots. Among the non-carbon support catalysts, tungsten carbide is relatively attractive due to its unique features. For the main plane of Pt and WC, the atomic arrangement is similar. Due to the relatively high density of electron states near the Fermi level and the influence of carbon, some W 5d electrons in WC become local electrons, so that WC has a Pt-like catalytic activity. WC is regarded as an electrocatalyst support for fuel cells due to its good electrochemical stability, high conductivity and synergism with a variety of metal-based catalysts, so the Pt/WC catalyst has a high intrinsic activity [11,12,13,14]. Chhina et al. [15] studied the electrochemical stability of Pt/WC and Pt/Vulcan XC-72. This work reported that WC used as a Pt support material could stabilize the surface of the catalyst and showed excellent corrosion resistance in an acid solution. Shen et al. [16,17] found that the oxygen reduction reaction activities of different noble metal catalysts would be enhanced when WC ameliorated the carbon support for these catalysts. Therefore, from the trend of these studies, WC is a suitable support material for fuel cell catalysts.

With continuous demands for hydrogen energy, many researchers have turned their attention to producing hydrogen with a water electrolysis strategy and the development of fuel cells. Interestingly, WC and the supported noble metal catalyst implies an excellent catalytic performance for the hydrogen evolution reaction (HER). In recent years, Pt and WC have been continuously reported as the main catalysts for HER. Ma et al. [18] studied the catalytic performance of Pt/WC-PME catalysts for HER, and found that the catalyst revealed good catalysis stability when the WC supported a nano-sized Pt catalyst. Lee et al. [19] tested tungsten carbide loaded with 7.5 wt.% of platinum for HER. The results indicated that the catalytic activity of the Pt/W_2_C catalyst was about 3 times more than a commercial 20 wt.% Pt/E-Tek catalyst. Shen et al. [20] revealed that a PtPd/WC/C catalyst could show higher activity than a Pt/WC/C catalyst in HER. According to the kinetic study, the PtPd/WC/C catalyst with the introduction of Pd has a high exchange current density and low overpotential in a 0.5 M H_2_SO_4_ solution. Esposito et al. [21] reported that a Pt/WC catalyst with the deposition of only one monolayer of Pt had the highest catalytic properties for HER. Although Pt/WC catalysts have been studied for many years, the particle size and structure of WC still limits its large-scale application as a Pt catalyst support for HER. Thus, it is important to make up a specific structure to improve the catalytic ability of Pt/WC catalysts.

In this work, nanoscale tungsten carbide as a support for a Pt catalyst was synthesized by solution combustion synthesis and a chemical reduction strategy. A uniform surface carbon layer was formed on the surface of tungsten carbide, which could fix Pt nanoparticles. By studying the distribution of amorphous carbon and Pt nanoparticles on the Pt/WC catalyst, the model diagram of the Pt/WC catalyst is obtained, which further illustrates the connections of Pt and WC catalysts. Compared with commercial Pt/C catalysts, the Pt/WC catalyst shows good electrochemical performance with an overpotential (η10) of 32.3 mV and a Tafel slope of 30 mV·dec^−1^ for HER, due to its excellent conductivity and structural stability. In addition, the coating carbon enhances the synergy between the Pt and WC catalysts, increasing the catalytic performance.

## 2. Experimental

### 2.1. Preparation of WC

The tungsten carbide was prepared by solution combustion and a chemical reduction strategy. In a typical synthesis, the raw materials consisted of ammonium tungstate ((NH_4_)_10_W_12_O_41_, 22.82 g, tungsten source), nitric acid (HNO_3_, 9.69 g, oxidant), urea (CO(NH_2_)_2_, 3 g, fuel) and glucose (C_6_H_12_O_6_·H_2_O, 15.34 g, carbon source) with a molar ratio of 3:40:20:31. Then the raw materials were added to 100 mL high-purity water, continuously stirring at room temperature. Next, the mixed solution was heated to 300 °C in a quartz electric furnace. During the heating process, the mixture produced a redox reaction of a highly exothermic self-contained combustion and evaporated into a shaggy particle like a gel. No carbonization occurred at this stage, and the as-synthesized product was named as the WC precursor. The WC precursor was transferred into a tubular furnace, and it was further reduced at 1100 °C in an Ar atmosphere for 6 h to obtain the WC nanoparticles.

### 2.2. Preparation of Pt/WC

In order to load Pt nanoparticles onto the WC surface, it was necessary to obtain a mixture of the WC precursor and Pt. Firstly, the WC precursor was ground into fine particles in a mortar. An amount of 0.95 g of WC precursor and 0.54 g of H_2_PtCl_6_·6H_2_O (Pt salt) were dispersed into 20 mL of deionized water. Then, the solution was adjusted to a pH of ~10 by adding 0.2 mol·L^−1^ NaOH solution. The mixture of the WC precursor and Pt salt was reduced by adding a CH_2_O (reductant) solution. Furthermore, the mixture was transferred into the oven to dry at 80 °C for 2 h after rinsing with high-purity water. Lastly, the mixture was transferred into a tubular furnace, and it was further reduced at 1100 °C in an Ar atmosphere for 6 h to obtain the Pt/WC nanoparticles. Based on the mass ratio of the WC precursor and Pt salt, the content of Pt in the Pt/WC catalyst was less than 20%. The commercial Pt/C catalyst (20 wt.% Pt, Alfa Aesar) was also used for comparison.

### 2.3. Characterization of Electrocatalysts

X-ray diffraction (XRD, Rigaku D/MAX-RB12) with Cu Kα radiation (λ = 1.5406 Å) was used to investigate the crystalline state and phase structure of the prepared Pt/WC catalyst, WC and WC precursor, and the scanning rate was set at 10° min^−1^. A field-emission scanning electron microscope (FESEM, Zeiss Ultra 55) was used to observe the particle size and morphology of the Pt/WC catalyst, WC and WC precursor. Transmission electron microscopy (TEM, JEOL, JEM-2010) was used to study the local structure and crystal structure. By using the energy dispersive spectroscopy (EDS), which coupled in SEM, the different elemental distribution and content of the Pt/WC catalyst were acquired. The near-surface chemical state and composition of different elements in the Pt/WC catalyst was also measured using X-ray photoelectron spectroscopy (XPS, ESCALAB 250 Xi).

### 2.4. Electrochemical Measurements

The catalytic performance measurements were implemented using an Ecochemie Autolab PGSTAT30 potentiostat at room temperature. Typically, the three-electrode test system was used. A saturated calomel electrode (SCE, (0.242 + 0.059 pH) V versus reversible hydrogen electrode (RHE)) was used as the reference electrode, a glassy carbon electrode (GCE, 3 mm in diameter) was used as the working electrode, and a Pt flag was used as the counter electrode. The surface of the GCE was polished with 0.05 µm Al_2_O_3_ slurry before using the glassy carbon electrode, and then the GCE was cleared by ultrasonic cleaning with high-purity deionized water. The mixed solutions were made by mixing 10 mg WC, commercial Pt/C and Pt/WC catalysts with 0.15 mL Nafion solution (D520, 5 wt.%), 0.35 mL ethanol and 1.5 mL deionized water, respectively. Furthermore, the mixture was ultrasonicated for 0.5 h, and 10 µL from each of the homogeneous dispersion solutions was injected into the electrode surface of the GCE. After drying for 8 h, the working electrode was ready for testing. Finally, the mass loading of catalyst was 0.7 mg·cm^2^ on the GCE surface during the film preparation process. The electrocatalytic activities of the Pt/WC, commercial Pt/C and WC catalysts for HER were evaluated by linear sweep voltammetry (LSV) curves at a sweep rate of 2 mV·s^−1^ in a 0.5 M H_2_SO_4_ solution. By fitting the obtained LSV curves, the Tafel plots of Pt/WC, commercial Pt/C and WC catalysts were obtained. To investigate the long-term stability of the Pt/WC catalyst, the electrode potentials were conducted from −0.2 to −0.51 V (vs. SCE, WC catalyst) and −0.2 to −0.31 V (vs. SCE, Pt/WC catalyst) in a 0.5 M H_2_SO_4_ solution at a scan rate of 50 mV·s^−1^.

## 3. Results and Discussion

### 3.1. Phase and Structural Characterization of WC and Pt/WC Catalysts

**Figure 1:**
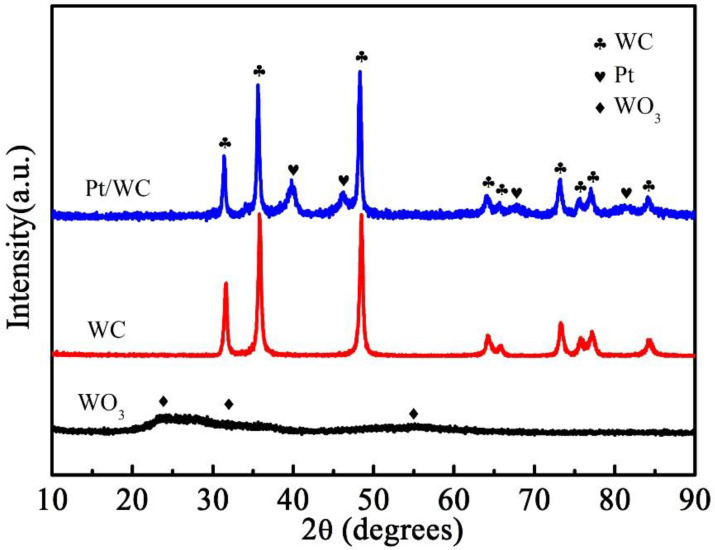
X-ray diffraction patterns of the WC precursor, WC and Pt/WC catalysts.

To determine the phase composition, the XRD patterns of the WC precursor, WC and Pt/WC catalysts are shown in Figure 1. The low-intensity diffraction peaks of the prepared WC precursor (Black line) were assigned to the WO_3_ phase [22], which indicates that the particles had a small size and low crystallinity. No obvious carbon peak makes clear the existence of amorphous carbon in the WC precursor during the solution combustion process. After high-temperature carbonization, according to the standard diffraction pattern (PDF = 02 − 0095), it was seen that the prepared product was WC (Red line). For the curve of WC nanoparticles in Figure 1, only strong WC diffraction peaks can be observed when the carbonization temperature was 1100 °C, indicating that the precursor had completely transformed into WC crystal. During the carbonization process, the Pt nanoparticles were successfully reduced on the WC surface. The crystalline Pt shows a face-centered cubic (fcc) structure, which is observed from the diffraction curve of Pt/WC catalyst (Blue line). The peaks at the 2θ of 39.8°, 46.4°, 67.5°, and 81.3° correspond to the (111), (200), (220) and (311) lattice planes of Pt, respectively. The diffraction peaks of WC and Pt suggest that the Pt nanoparticles were successfully loaded onto the WC supports.

**Figure 2:**
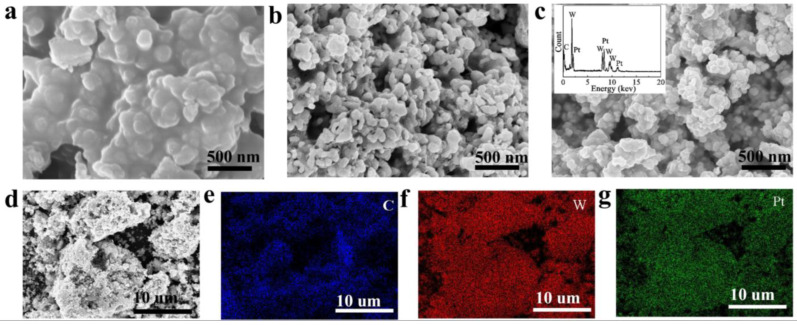
FESEM micrographs of: (**a**) WC precursor, (**b**) WC catalyst, (**c**) Pt/WC catalyst (inset of EDS spectrum), (**d**–**g**) element mapping images of the Pt/WC catalyst, including C, W and Pt elements.

The morphology and appearance of the WC precursor, WC and Pt/WC catalysts were observed by FESEM images, as shown in Figure 2. The FESEM micrographs of the WC precursor reveal that the small particles agglomerated into bulky grains, which is related to the fact that glucose was not completely reduced by the solution combustion reaction (Figure 2a). The shape of the WC powder is nearly spherical, while a few particles are elongated (Figure 2b). The nano-sized WC powders consisted of homogeneous particles with less than 100 nm average particle size, indicating that the reaction between WO_3_ and carbon results in particle refinement. Powders with small particle sizes have high specific surface area, which increases electrolyte access to the active fraction of the catalyst by lowering mass transport limitations. Figure 2c shows the micrographs and particle sizes of the Pt/WC catalyst. Compared with WC, the big particle size of the Pt/WC catalyst was ascribed to the carbonization process in which the introduction of Pt enhanced the growth of WC particles. The presence of C, W and Pt elements was exhibited via EDS spectra (inset of Figure 2c). The multifarious elements content of the Pt/WC catalyst is shown in Table 1. Figure 2d reveals that carbon, tungsten and platinum elements were homogenously distributed in the Pt/WC catalyst, according to the FESEM elemental mapping analysis. According to previous research in our group, the carbon content of prepared WC is 7.1 wt.%, which is higher than the theoretical carbon content (C/WC=6.1 wt.%), suggesting that the extra carbon exists in the Pt/WC catalyst [23]. Additionally, the emergence of oxygen content in the EDS analysis was generated due to the oxidation of W when the WC was exposed to the air [24].

**Figure 3:**
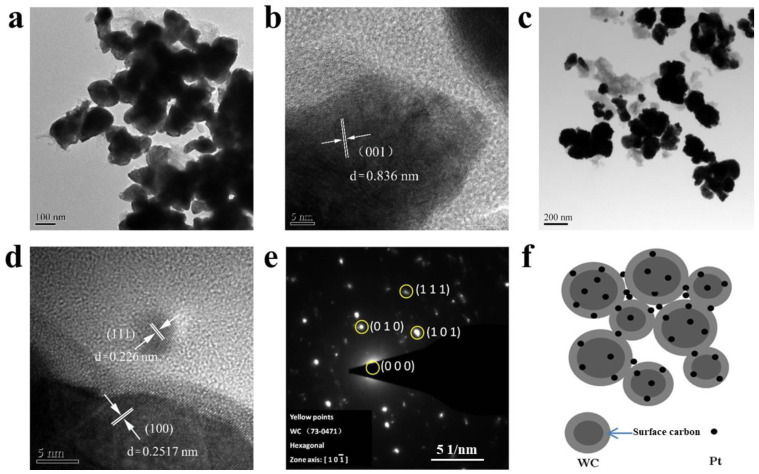
Characterization of WC and Pt/WC catalysts: (**a**,**b**) TEM images of WC; (**c**,**d**) TEM images of Pt/WC catalyst; (**e**) SAED pattern of Pt/WC catalyst; (**f**) construction of Pt/WC catalyst.

The microstructure and local region of the synthesized Pt/WC and WC catalysts were further analyzed by TEM images (Figure 3). A wide particle size of the WC catalyst can be observed and particle agglomeration occurred during the high-temperature carbonization (Figure 3a,b). The interplanar distance on the surface of the WC nanoparticles was measured at 0.836 nm, which corresponds to the (001) plane, indicating that the WC had a good crystallinity. Figure 3c,d show the magnified TEM images of the Pt/WC catalyst. The lattice fringe of the particles in the Pt/WC catalyst corresponds to the interplanar distances of 0.2517nm and 0.226nm, respectively (Figure 3d). Those agree well with the (100) plane of W and the (111) plane of Pt. It is clear that Pt nanoparticles with a diameter less than 5 nm were scattered on the surface carbon. A contact carbon layer between Pt and WC particles is very important for the enhancement in catalytic activity via a synergism for HER [25]. The polycrystalline nature of the Pt/WC catalyst is proved by the selected-area electron diffraction (SAED) pattern (Figure 3e). The HRTEM image shows the diffraction pattern corresponding to the [10$\bar{1}$] zone axis of WC by the measurement of reciprocal lattice distances and intersection angles of the SAED pattern. Figure 3f shows the construction of the Pt/WC catalyst nanostructures. There was a thin carbon layer on the surface of the WC particles. Pt nanoparticles can be evenly dispersed and fixed on this carbon layer. Carbon was found to be an important influencing factor for the electrocatalytic properties of WC in a previous study [26]. Owing to the extra carbon coating on the surface of the prepared WC, it is helpful to improve the electrical conductivity and structural stability between the Pt and WC catalysts, so that the catalytic performance of the composite catalysts will be indeed improved.

**Figure 4:**
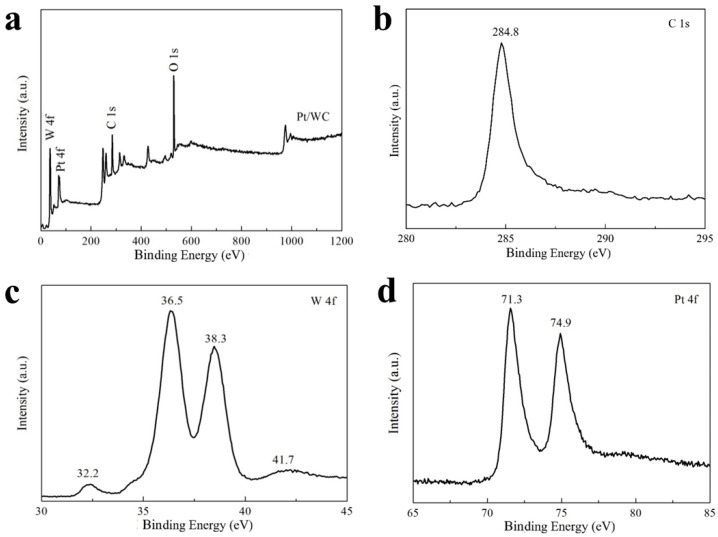
(**a**) XPS spectra of Pt/WC catalyst; (**b**) C 1s peak; (**c**) W4f peak; (**d**) Pt 4f peak.

XPS curves were used to further understand the element valence state and composition on the surface of the Pt/WC catalyst. The full XPS spectra shows the existence of oxygen, tungsten, carbon, and platinum on the Pt/WC catalyst, as shown in Figure 4a. To avoid the influence of test error, the spectrum was calibrated using the standard C 1s signal located at 284.8 eV (Figure 4b). Figure 4c shows the W 4f XPS spectra of the Pt/WC catalyst, which shows that the photoemission spectrum of W 4f was composed of four distinct peaks. The W 4f peaks with 32.2 eV and 36.5 eV binding energies are assigned to WC, while the other peaks with 38.3 eV and 41.7 eV binding energies correspond to WO_x_. The considerable proportion of WO_x_ may be attributed to the exposure of the Pt/WC catalyst to the atmosphere. According to previous literature reports, the presence of some oxygen vacancies on the surface of WC is conducive to improving its catalytic performance for HER [27]. The spectrum of Pt in the Pt/WC catalyst consisted of two peaks with 71.3 eV and 74.9 eV binding energies (Figure 4d), which respectively corresponds to Pt and PtO_2_. This result also proves that Pt nanoparticles were generated in the carbonization stage and exist on the surface of the WC/C. The XPS spectra reflect that the Pt/WC catalyst consists of WO_x_, WC, platinum and carbon, which is consistent with the EDS measurements.

### 3.2. Electrochemical Activity of Different Catalysts

**Figure 5:**
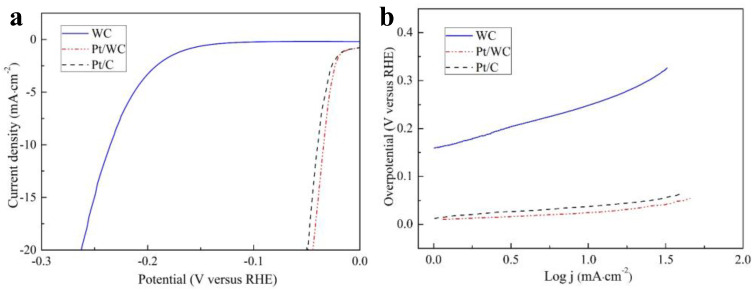
HER performances of WC, commercial Pt/C and Pt/WC catalysts in 0.5 M H_2_SO_4_ solution: (**a**) Polarization curves at 2 mV·s^−1^; (**b**) Tafel plots by fitting LSV curves.

Three-electrode measurement was used to study the catalytic performance of the different catalysts at a scanning rate of 2 mV·s^−1^ in a 0.5 M H_2_SO_4_ solution. The catalytic ability of the commercial 20% Pt/C catalyst was also tested to compare with the prepared Pt/WC and WC catalysts. Figure 5 shows the representative polarization curves and Tafel plots of the Pt/WC, Pt/C and WC catalysts. Usually, the overpotential of different catalysts at a current density of 10 mA·cm^−2^ is denoted as η10, which is used to evaluate the HER activities. For the WC electrode, the η10 was -238.3 mV (Figure 5a). The polarization curve of the WC catalyst indicates that it has a good catalytic performance for HER. So WC can be considered as an effective Pt support material, which could decrease the consumption of Pt and improve its electrochemical performance. Interestingly, the Pt-like activity could be produced by modifying the WC surface with platinum, and the similar activity of HER is due to the similar electronic properties of bulk Pt and Pt nanoparticles on WC [28]. The as-synthesized Pt/WC catalyst exhibited a more positive η10 of 32.3 mV and a higher current density than the Pt/C catalyst, testifying that the synergistic mechanism between Pt and WC catalysts has an important effect on the HER activity. The extra carbon layer between the Pt and WC nanoparticles improves the conductivity of the whole catalyst and allows Pt nanoparticles to be evenly distributed and fixed. Then, the improvement in the structural stability of catalysts is conducive to the improvement of electrochemical stability. In order to get more information on HER, a Tafel slope is obtained by fitting the LSV curves of different catalysts. As an intrinsic characteristic of fuel cell catalysts, the Tafel slope is identified as an indicator involving electron transfer for various catalytic reactions. The exchange current density can be obtained via the relative intercept. The relevant data values are presented in Table 2. As shown in Figure 5b, the Tafel slope and the exchange current density of the Pt/WC catalyst are 30 mV·dec^−1^ and 0.829 mA·cm^−2^, respectively. For the pure WC catalyst, the Tafel slope is 88 mV·dec^−1^. The small value of the Tafel slope means that the Pt/WC catalyst has the highest catalytic activity. Owing to the existence of surface carbon on the WC and nanoscale catalyst, which increases the solution’s access to the active fraction of Pt/WC, the reaction kinetics of HER are further improved [29]. These results prove that the support materials have an important influence on the Pt catalyst.

**Figure 6:**
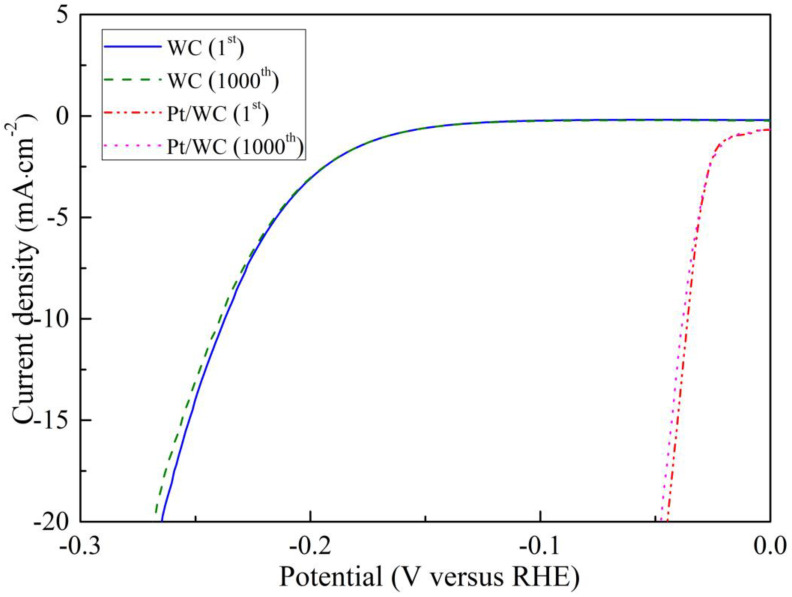
Polarization curves of WC and Pt/WC catalysts after continuous potential sweeps at 50 mV·s^−1^ in 0.5 M H_2_SO_4_ solution.

To study the durability of the Pt/WC and WC catalysts in the hydrogen evolution reaction, the prepared electrodes were cycled over 1000 cycles in a 0.5 M H_2_SO_4_ solution, and the scanning rate was set at 50 mV·s^−1^. Figure 6 exhibits the polarization curves of the WC and Pt/WC catalysts in an acidic condition. Only a slight deterioration of the cathodic current density was observed after 1000 cycles, demonstrating that the Pt/WC and WC catalysts possess excellent cycling stability. Compared with the catalytic influence of the WC catalyst, Pt loaded by WC can maximize its role. WC not only possesses a catalytic effect by itself, but also acts as a noble metal support. For the Pt/WC electrode, tungsten carbide can enhance the corrosion resistance of the Pt/WC catalyst for its good electrochemical stability. Nano-sized WC with a large specific surface area could facilitate mass transport and provide more active sites, which is conducive to the adsorption of hydrogen atoms. In addition, the carbon layer on the surface of WC can further improve its electroconductivity and catalytic stability. By loading Pt onto WC, the amount of platinum is also greatly reduced [30]. Therefore, it is reasonable to believe that WC can be considered as an ideal Pt catalyst support candidate.

## 4. Conclusions

As a support of Pt catalysts, nanoscale tungsten carbide was prepared by improved solution combustion and a high-temperature carbonization strategy. The Pt nanoparticles were uniformly distributed on the external surface of WC nanoparticles after a carbonation process. The carbon on the surface of the tungsten carbide connected WC and Pt. The excellent performance of the Pt/WC catalyst is mainly attributed to the homogenous nano-sized tungsten carbide with the surface carbon. The small particle size of the Pt/WC catalyst accelerates electrolyte access to the active fraction of the catalyst by lowering mass transport limitations. The extra carbon coating on the surface of the prepared WC is helpful to stabilize the Pt nanoparticles and improve the conductivity of the whole catalyst. Moreover, the carbon layer could improve the synergy between Pt and WC, and the catalytic performance was indeed improved. The Pt/WC catalyst showed a positive η10 of 32.3 mV and a high current density, and the Tafel slope was 30 mV·dec^−1^. After an electrochemical durability test, the Pt/WC catalyst exhibited good electrochemical stability after 1000 cycles.

## Figures and Tables

**Table 1:** The element content of the Pt/WC catalyst via EDS analysis.

Element	Weight%	Atomic%
C	15.04	63.07
O	4.88	15.36
W	57.09	15.64
Pt	22.99	5.93
Totals	100.00	100.00

**Table 2:** HER activities of different catalysts from LSV measurement in 0.5 M H_2_SO_4_ solutions at room temperature.

Catalysts	Onset η (mV versus RHE)	η at j = 10 mA·cm^−2^ (mV versus RHE)	Tafel Slope (mV per Decade)	Exchange Current Density (mA·cm^−2^)
WC	136	238.3	88	0.202
Pt/WC	10	32.3	30	0.829
Pt/C	12	37.3	32	0.772

## Data Availability

The data presented in this study are available on request from the corresponding author.
